# Mobility decline in patients hospitalized in an intensive care unit

**DOI:** 10.5935/0103-507X.20160025

**Published:** 2016

**Authors:** Fábio Santos de Jesus, Daniel de Macedo Paim, Juliana de Oliveira Brito, Idiel de Araujo Barros, Thiago Barbosa Nogueira, Bruno Prata Martinez, Thiago Queiroz Pires

**Affiliations:** 1 Hospital Santo Antonio, Obras Sociais Irmã Dulce - Salvador (BA), Brazil.; 2 Universidade do Estado da Bahia - Salvador (BA), Brazil.; 3 Reative Fisioterapia Especializada - Salvador (BA), Brazil.

**Keywords:** Mobility limitation, Inpatients, Mortality, Morbidity, Intensive care units

## Abstract

**Objective:**

To evaluate the variation in mobility during hospitalization in an intensive care unit and its association with hospital mortality.

**Methods:**

This prospective study was conducted in an intensive care unit. The inclusion criteria included patients admitted with an independence score of ≥ 4 for both bed-chair transfer and locomotion, with the score based on the Functional Independence Measure. Patients with cardiac arrest and/or those who died during hospitalization were excluded. To measure the loss of mobility, the value obtained at discharge was calculated and subtracted from the value obtained on admission, which was then divided by the admission score and recorded as a percentage.

**Results:**

The comparison of these two variables indicated that the loss of mobility during hospitalization was 14.3% (p < 0.001). Loss of mobility was greater in patients hospitalized for more than 48 hours in the intensive care unit (p < 0.02) and in patients who used vasopressor drugs (p = 0.041). However, the comparison between subjects aged 60 years or older and those younger than 60 years indicated no significant differences in the loss of mobility (p = 0.332), reason for hospitalization (p = 0.265), SAPS 3 score (p = 0.224), use of mechanical ventilation (p = 0.117), or hospital mortality (p = 0.063).

**Conclusion:**

There was loss of mobility during hospitalization in the intensive care unit. This loss was greater in patients who were hospitalized for more than 48 hours and in those who used vasopressors; however, the causal and prognostic factors associated with this decline need to be elucidated.

## Abstract (pt)

**Objetivo:**

Avaliar a variação da mobilidade durante a internação em unidade de terapia intensiva e sua associação com mortalidade hospitalar.

**Métodos:**

Estudo prospectivo realizado em uma unidade de terapia intensiva. Os critérios de inclusão foram pacientes admitidos com escore de independência para transferência cama-cadeira e locomoção ≥ 4 cada um, baseado na escala Medida de Independência Funcional. Foram excluídos aqueles pacientes que apresentaram parada cardiorrespiratória e/ou evoluíram a óbito durante a internação. Para mensuração da perda de mobilidade calculou-se o valor obtido na alta, subtraído daquele obtido na admissão, o qual foi posteriormente dividido pelo escore da admissão e registrado em porcentual.

**Resultados:**

Na comparação dos dois domínios avaliados, foi observada perda de mobilidade durante a internação de 14,3% (p < 0,001). A perda foi maior nos pacientes internados por mais de 48 horas na unidade (p < 0,02) e naqueles que usaram drogas vasopressoras (p = 0,041). Não houve diferença na perda de mobilidade na comparação das variáveis paciente idoso (p = 0,332), motivo de internação (p = 0,265), SAPS 3 (p = 0,224), uso de ventilação mecânica (p = 0,117) e óbito (p = 0,063).

**Conclusão:**

Houve declínio de mobilidade durante a internação na unidade de terapia intensiva. Este foi maior nos pacientes que ficaram mais que 48 horas na unidade e nos que usaram drogas vasopressoras, sendo necessária a identificação dos fatores causais e prognósticos deste declínio.

## INTRODUCTION

Mobility decline is defined as the partial or total loss of the ability to perform activities of daily living, including transfer to bed, transfer from bed to chair, and locomotion.^(1)^ This loss is referred to as functional decline; however, this term does not fit the requirements of the International Classification of Functioning, Disability, and Health (ICF).^(2,3)^ Understanding these changes is relevant to health professionals because of the possible complications of inactivity and the possibility of developing preventive interventions to minimize these complications, particularly in intensive care units (ICUs).

The limited capacity to evaluate the predictable loss of mobility in patients in ICUs can be explained by the inability to measure this condition at the time of admission. Therefore, one of the strategies used to measure the variation in mobility during ICU stay is the comparison of the values obtained at ICU discharge from independence values obtained 48 hours before admission to the ICU.^(3)^

The attempts to minimize this decline is a goal of the multidisciplinary team because reduced mobility is associated with several negative outcomes, including sarcopenia,^(4)^ falls,^(5)^ and even death in older adults.^(6)^ Therefore, considering that many patients have preserved mobility before ICU admission and that hospitalization in the ICU may predispose these patients to increased immobility for different reasons, the present study aimed to evaluate the variation in mobility status before hospitalization and at ICU discharge and whether this variation was associated with hospital mortality.

## METHODS

This prospective study was conducted in the ICU of the *Hospital Santo Antônio* of the *Obras Sociais Irmã Dulce* between January and October 2013 to assess the variation in mobility status during hospitalization in this unit. This ICU has a general profile and assists clinical and surgical patients (particularly patients who have undergone abdominal surgery). This study was approved by the Research Ethics Committee of the *Hospital Santo Antônio* under protocol no. 399278/2013. All those in charge of the patients were informed about the study and signed an informed consent form to authorize the patients' participation in the study.

The patients included were those admitted to the ICU who had independence scores measured before admission of ≥ 4 for both bed-to-chair transfer and locomotion. Patients who had cardiac arrest or died during the ICU stay were excluded.

The scale used to measure the degree of independence before admission was the Functional Independence Measure (FIM) scale, and the domains of bed-to-chair transfer and locomotion were used.^(7)^ This scale measures the ability to perform activities of daily living in addition to measuring cognitive functions. Some of the motor tasks measured are bed-to-chair transfer and ambulation, which were used in this study. The initial measurements were made by the unit's physical therapist during patient admission to the ICU, and the mobility status of the patients 48 hours before admission was used because most patients were not clinically stable to undergo the actual measurements. The mobility status was determined with the help of the family in cases in which the patients could not report the status. The second measurement was made upon discharge from the ICU, and at this time, the variables proposed in the FIM were measured when the patients performed mobility activities with the greatest possible independence, and assistance was provided only in cases of extreme necessity.

The score for each domain varied between 1 and 7. On the Likert scale, the value of 1 indicated total dependence to perform activities of daily living, and 7 indicated complete independence.^(7)^ Loss of mobility was measured during the ICU stay by subtracting the mobility value obtained at discharge from the value obtained at admission, dividing it by the score at admission, and recording it as a percentage. Other variables recorded during the study period included the Simplified Acute Physiology Score 3 (SAPS 3) severity score,^(8)^ reason for admission to the ICU (medical or surgical), length of stay, use of vasoactive drugs (vasopressors), use of invasive mechanical ventilation, use of hemodialysis, and hospital mortality. Patients aged ≥ 60 years were classified as older adults and were compared with patients aged < 60 years.

It is important to note that the ICU had physiotherapists available 24 hours a day, and the focus was the early mobility of hospitalized patients, per the recommendations of the literature and the safety criteria for performance of physical therapy activities.^(9,10)^ The activities performed included neuromuscular electrostimulation of the lower limbs, global kinesiotherapy, physical activity training (transfer from bed to chair, sitting position with the legs not touching the floor., orthostasis, and ambulation), and footboard. The activities were usually performed in the morning and afternoon and lasted between 20 and 40 minutes depending on the complexity of each case.

The sample size was calculated using a standard deviation of 4 points, a difference to be detected of 2 points in the period from 48 hours before admission until discharge, a level of significance of 95%, and study power of 80%, which resulted in a sample size of 63 patients. The variables with abnormal distribution, such as length of stay and SAPS 3 score, are described as medians and interquartile ranges. To compare the variables bed-to-chair transfer and locomotion between admission and discharge, we used the nonparametric Wilcoxon's test for paired samples because the data had a non-normal distribution. For comparison of the percentage of loss of mobility for the variables length of ICU stay (≤ 48 hours and > 48 hours), age ≥ 60 years (older adults), reason for hospitalization, use of mechanical ventilation, use of vasoactive drugs, hospital mortality, and SAPS 3 score ≥ 57, the Mann-Whitney test for unpaired samples was used. A p-value < 0.05 was considered significant. All analyses were performed using the Statistical Package for Social Sciences (SPSS) version 14.0.

## RESULTS

**Figure 1:**
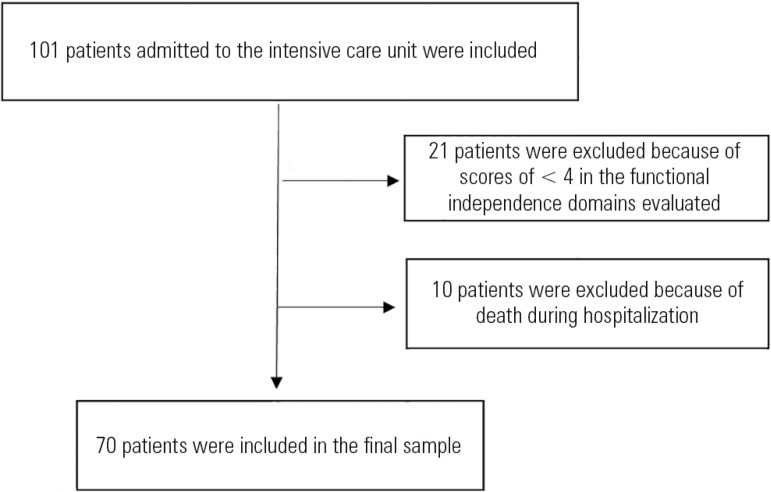
Flowchart of patient selection.

Of the 101 patients enrolled in the study, 31 were excluded (Figure 1). The final sample consisted of 70 patients with a mean age of 56.7 ± 3.4 years. With regard to the reasons for hospitalization, there was a predominance of surgical cases, particularly abdominal surgeries, followed by sepsis, congestive heart failure, and decompensated cirrhosis (Table 1).

**Table 1:** General characteristics of the study sample (N = 70)

**Variable**	**Median (25% - 75%)**	**N (%)**
SAPS 3	38.5 (29.0 - 58.3)	
Length of stay in the ICU	2.0 (1.0 - 4.0)	
Gender		
Male		33 (47.1)
Female		37 (52.9)
Profile upon ICU admission		
Surgical case		48 (68.6)
Clinical case		22 (31.4)
Older adults		35 (50.0)
Reason for hospitalization		
Abdominal surgery		43 (61.4)
Sepsis		9 (12.9)
Congestive heart failure		5 (7.1)
Liver cirrhosis		5 (7.1)
Decompensated diabetes mellitus		2 (2.9)
Head and neck surgery		4 (5.6)
Vascular surgery		1 (1.4)
Neoplasms		1 (1.4)
Use of vasoactive drugs		20 (28.6)
Use of mechanical ventilation		23 (32.9)
Use of hemodialysis		5 (7.1)
Hospital mortality		11 (15.7)

SAPS 3 - Simplified Acute Physiology Score 3; ICU - intensive care unit.

With regard to the variables bed-to-chair transfer and locomotion, there was a significant decline of 14.3% between admission and discharge from the ICU (Table 2). The comparison of these two variables between patients hospitalized for < 48 hours and those hospitalized for ≥ 48 hours indicated a greater mobility decline in the locomotion domain among those who stayed longer in the ICU (p = 0.007) and among those who used vasopressors (p = 0.041) (Table 3). The comparison between subjects aged ≥ 60 years and those aged < 60 years indicated no significant difference in mobility decline during ICU stay (p = 0.332), cause for hospitalization (p = 0.265), SAPS 3 score (p = 0.224), use of mechanical ventilation (p = 0.177), or hospital mortality (p = 0.223).

**Table 2:** Functional independence scores for bed-to-chair transfer and locomotion domains between admission and discharge from the ICU

**Domain**	**Admission**	**Discharge**	**p-value**
Bed-to-chair transfer	7 (6 - 7)	6 (5 - 7)	0.001*
Locomotion	7 (6 - 7)	6 (4 - 7)	0.001*
Total score	14 (12 - 14)	12 (10 - 14)	0.001*

* 50% interquartile range (25% - 75%).

**Table 3:** Scores for the loss of mobility considering different lengths of stay in the intensive care unit and the use of vasopressors

**Domain**	**Loss (mean rank)**		**p-value**
	**LS ≤ 48 hours**	**LS > 48 hours**	
Bed-to-chair transfer	30.3	41,1	0.015
Locomotion	29.7	41.9	0.007
Total score	29.7	41.9	0.007
	Vasopressors (yes)	Vasopressors (no)	
Bed-to-chair transfer	29.8	38.4	0.054
Locomotion	29.5	39.0	0.038
Total score	29.6	40.0	0.041

LS - length of stay.

## DISCUSSION

In addition to supporting treatment of the disease and ensuring the survival of patients in the ICU, the multidisciplinary team should not underestimate the capacity of mobility of patients during hospitalization. The ICU stay is associated with risk factors for greater morbidity, including the decreased ability to perform activities of daily living, because of the positive correlation between periods of immobility secondary to acute clinical conditions and other factors, including the use of sedatives and vasoactive drugs, use of catheters, and renal replacement therapy, all of which limit mobility.^(11-13)^ This study is one of the few to evaluate the variation in mobility during the ICU stay, and this factor has a strong correlation with human movement.^(14)^ Most other published studies only evaluated the mobility status of patients at ICU discharge and compared it with the status at or after hospital discharge; these evaluations differ from those of our study.

The predominance of surgical cases in the study sample may explain the 14.3%, reduction in mobility, which was lower than that found in a study that evaluated functionality between hospital admission for cardiac surgery and the post-operative period (18%).^(15)^ This percentage was also lower than values found in other studies, such as that of Martinez et al.,^(2)^ who reported a loss of mobility of 25.9% in the period between ICU admission and discharge, and that the study of Covinsky et al.,^(11)^ who reported a mobility loss of 35% between hospital admission and discharge. It is of note that the latter study had a predominance of clinical cases,^(11)^ which differs from the results of the present study and the study by Martinez et al.^(2)^

Another crucial factor in patients hospitalized in the ICU is age, as older age is strongly correlated with lower functionality and worse outcomes.^(16,17)^ Our study found no significant difference in mobility loss in older adults, which is probably because of the lack of significant differences between the SAPS 3 score and the length of stay in the ICU. Moreover, the older population receives increased care from the multidisciplinary team because it has a higher risk for sarcopenia, which is a public health problem associated with higher mortality.^(4,6)^

Covinsky et al.^(11)^ reported a functional decline of 50% in patients older than 85 years, and this result was attributed to the greater number of chronic degenerative diseases in this age group. Siqueira et al.^(16)^ reported that hospitalization in older adults is a high-risk factor, and the prognosis is worse in the presence of two or more chronic diseases, considering the complications caused by these diseases and the extended period these patients need to recover. The number of associated pathologies seems to be a determinant of clinical outcomes of patients in the ICU;^(18)^ however, the impact of comorbidities on the functional status of patients has only been investigated recently. In addition, many factors can influence mobility decline, particularly inactivity, malnutrition, and factors that do not involve patients, including cultural factors of the multidisciplinary team, who do not prioritize mobility in patients, despite the positive outcomes of increased mobility found in the literature.^(9,10)^

The increased loss of mobility observed in the group hospitalized for more than 48 hours in the ICU may be explained by their greater exposure to factors that can restrict mobility, although all the patients evaluated ambulated daily. Similarly, Martinez et al. found increased loss of mobility in patients hospitalized for more than 48 hours.^(2)^

Although the multidisciplinary team prioritizes the factors associated with reduction of hospital mortality, patient mobility upon ICU discharge is associated with lower morbidity and can foster greater social reintegration and possibly lower the risk of readmissions in specific groups.^(19,20)^ In this context, the focus on minimizing the risk of mobility loss is essential during hospitalization, particularly in populations at risk, and this risk should be assessed using reliable instruments. Nevertheless, there was no association between loss of mobility and hospital mortality despite mobility being related to morbidity; mortality is related to systemic problems, which may not necessarily be related to human movement. In addition, the difference in the loss of locomotor mobility in patients who used vasopressors was probably due to locomotor limitations secondary to septic shock, although no difference in the bed-to-chair transfer was observed.

The study has some limitations, including the lack of control of other confounding factors, such as the use of medications and the occurrence of hyperglycemia, which can influence mobility decline. However, our study aimed to measure the probable mobility decline; for this reason, further investigation is necessary to elucidate the factors associated with mobility loss. Another important aspect was the use of a non-specific instrument for evaluating mobility in the ICU, which allowed the assessment of only two variables (bed-to-chair transfer and locomotion). For this reason, measurement bias may have occurred during the reporting of the mobility status before admission to the ICU; however, considering that the mobility of most patients was preserved before admission, this limitation was minimized.

## CONCLUSION

There was mobility decline during hospitalization in the intensive care unit with respect to bed-to-chair transfer and locomotion. This decline was greater in patients hospitalized for more than 48 hours in the intensive care unit and in those who used vasopressors; however, the causal and prognostic factors associated with this decline need to be identified.
